# Early Identification of Pneumonitis in Patients Irradiated for Lung Cancer—Final Results of the PARALUC Trial

**DOI:** 10.3390/cancers15020326

**Published:** 2023-01-04

**Authors:** Dirk Rades, Elisa M. Werner, Esther Glatzel, Sabine Bohnet, Steven E. Schild, Søren S. Tvilsted, Stefan Janssen

**Affiliations:** 1 Department of Radiation Oncology, University of Lubeck, 23562 Lubeck, Germany; 2 Department of Pulmonology, University of Lubeck, 23562 Lubeck, Germany; 3 Department of Radiation Oncology, Mayo Clinic, Scottsdale, AZ 85259, USA; 4 Research Department, Zealand University Hospital, 4600 Køge, Denmark; 5 Medical Practice for Radiotherapy and Radiation Oncology, 30161 Hannover, Germany

**Keywords:** lung cancer, radiotherapy, pneumonitis, patient reported outcomes, symptom-based scoring system, mobile application

## Abstract

**Simple Summary:**

Pneumonitis is a serious complication following radiotherapy for lung cancer. Since it generally occurs weeks or even months following treatment, it is often not attributed to the previous irradiation and may be missed. The PARALUC trial was performed to help develop a mobile application (app) that allows early diagnosis and treatment of radiation pneumonitis. The primary goal was the identification of the optimal cut-off of a pneumonitis score (presence or absence of pneumonitis). Based on the severity of related symptoms, scoring points ranged between 0 and 9. The highest sensitivity was achieved with 0–4 points, followed by 5 points, and the highest specificity with 5–6 points. The highest Youden-index (optimal cut-off) was found for 5 points. Moreover, pneumonitis was significantly associated with an increase of ≥3 points from baseline. Patient satisfaction with this scoring tool was very high. Five points were the optimal cut-off to differentiate between pneumonitis and other pulmonary morbidities. The score provided high diagnostic accuracy and patient satisfaction.

**Abstract:**

Radiotherapy of lung cancer may cause pneumonitis that generally occurs weeks or months following therapy and can be missed. This prospective trial aimed to pave the way for a mobile application (app) allowing early diagnosis of pneumonitis. The primary goal was the identification of the optimal cut-off of a score to detect pneumonitis of grade ≥2 after radiotherapy for lung cancer. Based on the severity of symptoms (cough, dyspnea, fever), scoring points were 0–9. Receiver operating characteristic (ROC)-curves were used to describe the sensitivity and specificity. The area under the ROC-curve (AUC) was calculated to judge the accuracy of the score, Youden-index was employed to define the optimal cut-off. Until trial termination, 57 of 98 patients were included. Eight of 42 patients evaluable for the primary endpoint (presence or absence of radiation pneumonitis) experienced pneumonitis. AUC was 0.987 (0.961–1.000). The highest sensitivity was achieved with 0–4 points (100%), followed by 5 points (87.5%), highest specificity with 5–6 points (100%). The highest Youden-index was found for 5 points (87.5%). The rate of patient satisfaction with the symptom-based scoring system was 93.5%. A cut-off of 5 points was identified as optimal to differentiate between pneumonitis and no pneumonitis. Moreover, pneumonitis was significantly associated with an increase of ≥3 points from baseline (*p* < 0.0001). The scoring system provided excellent accuracy and high patient satisfaction. Important foundations for the development of a mobile application were laid.

## 1. Introduction

Many patients with non-small-cell lung cancer (NSCLC) or small-cell lung cancer (SCLC) receive radiotherapy alone, or more frequently, combined with systemic therapy [1]. Treatment may lead to pneumonitis, which can be debilitating for patients and fatal in 2% of the cases [2]. Pneumonitis generally develops weeks or months after radiotherapy is completed [3,4]. Therefore, pneumonitis can be missed, if the symptoms including cough, dyspnea, and fever are not put into context with the radiotherapy administered in the recent past [5]. In order to rapidly provide appropriate treatment, it is important to diagnose radiation pneumonitis early. Identification of patients with pneumonitis would be facilitated with a mobile application (app) that the patients can use at home. Such an app may provide a score correlating with the probability of pneumonitis. However, prior to an introduction to the health market, certain prerequisites need to be fulfilled. For example, the optimal cut-off regarding the scoring points in terms of sensitivity and specificity should be defined. Moreover, the survey tool must be rated usable by patients.

The major goals of this prospective trial were to develop a symptom-based score identifying the optimal cut-off regarding the scoring points to identify patients with symptomatic pneumonitis grade ≥2 according to Common Terminology Criteria for Adverse Events version 5.0 (CTCAE v5.0 [6]) and describe patients’ satisfaction with the scoring system. If the study was successful in identifying the optimal scoring point for the detection of symptomatic pneumonitis and patient dissatisfaction rates were low, an app could be developed and tested in further prospective trials. A version of the study protocol without results was described previously [7]. 

## 2. Materials and Methods

The prospective interventional trial was approved by the responsible local ethics committees in Lübeck and Hannover (leading committee at the University of Lübeck, reference 20-025), registered at clinicaltrials.gov (identifier: NCT04335409), and conducted in accordance with the Declaration of Helsinki. The inclusion criteria were histologically proven lung cancer, local or loco-regional irradiation, patient age of at least 18 years, written informed consent, the capacity of the patient to contract and cooperate, and risk factors for radiation pneumonitis, i.e., a mean radiation dose to ipsilateral lung > 20 Gy or a mean dose > 13 Gy, plus at least one other risk factor including significant cardiovascular disease, a history of heavy smoking (≥40 pack years), and chemo- or -immunotherapy prior or during the radiotherapy course [3,8,9,10]. Exclusion criteria were pregnancy or lactation, expected non-compliance, and (after an amendment of the study protocol in August 2021) a baseline score of >3 points, as these patients will likely not be able to tolerate the planned treatment with full-dose radiation. During the course of radiotherapy, patients were seen by a physician once a week and asked to rate the degree of symptoms potentially associated with pneumonitis, i.e., cough, dyspnea, and fever (Table 1). Following radiotherapy, patients were contacted by phone (to reduce the number of visits to the hospital, particularly during the COVID-19 pandemic) once a week for 24 weeks or until the individual end of the study. Based on the severity of symptoms, individual scores for the patients ranged between 0 and 9 points (Table 1). If the individual score increased by 2 points compared to baseline, patients were seen by a pulmonologist. In case of suspected pneumonitis, further diagnostic procedures including lung function tests and, if indicated, diagnostic imaging were performed. The diagnosis of pneumonitis (vs. infection) was based on bronchoalveolar lavage fluid and microbiological findings. The diagnosis of radiation-induced pneumonitis was made if ground-glass opacities, consolidation, or both confined to the radiation fields were found on computed tomography scans [11,12]. If the diagnosis of pneumonitis grade ≥ 2 (according to CTCAE v5.0 [6]) was confirmed, patients received corticosteroids [4,10].

According to the study protocol, the primary goal of this trial was to assess the performance characteristics of a symptom-based scoring system for the detection of radiation pneumonitis during or following radiotherapy for lung cancer [7]. The receiver operating characteristic (ROC) curve was used to show the connection between sensitivity and specificity for every possible cut-off for the scoring system and to select the optimal scoring point for the detection of radiation pneumonitis. The area under the ROC curve (AUC) was calculated to prove the diagnostic ability of the scoring system [7]. Due to medical devices and data protection regulations, the study used a paper-based symptom scoring system instead of a computer app. In addition, the maximum increase of the scores during the study period when compared to baseline scores was assessed and compared between patients with and without pneumonitis (Fisher’s exact test). Other secondary endpoints included positive (PPV) and negative (NPV) predictive values associated with each scoring point of the scoring system. PPV was defined as the probability that patients with higher numbers of points develop pneumonitis, and NPV as the probability that patients with low numbers do not experience pneumonitis.

The PPV to correctly predict pneumonitis was calculated with:

PPV = [patients with pneumonitis/(patients with pneumonitis + without pneumonitis)] × 100

The NPV to correctly predict no development of pneumonitis was calculated with:

NPV = [patients without pneumonitis/(patients without pneumonitis + with pneumonitis)] × 100

Thus, the predictive values describe the performance of the scoring system and the value for the patients, whereas sensitivity and specificity describe the intrinsic validity of the test criterion. Another secondary endpoint was patient satisfaction with the symptom-based scoring system, which was assessed at the end of the radiotherapy course using a questionnaire modified according to Schrepp et al. [13]. Patients were asked to rate on separate scales ranging from 1 to 7 points, whether they found the symptom-based scoring system comprehensible, supportive, and safe. One point was given if the patients found the scoring system very incomprehensible, felt very disturbed by being asked to use the scoring system and rate their symptoms every week, and felt very insecure with the scoring system in general, respectively. Seven points were given if the patients found the scoring system very comprehensible, considered it very supportive when stating and rating their symptoms, and felt very safe with the scoring system in general, respectively. Thus, higher scores meant higher degrees of satisfaction. Mean values plus standard deviations were calculated for each aspect and each patient. A patient with a mean value of <4.0 was considered not satisfied. In case of a dissatisfaction rate > 20%, the scoring system was considered to require modifications before further use. If the dissatisfaction rate was >40%, the scoring system was considered not useful for further studies.

In addition, several patient and tumor factors were analyzed for associations with the development of pneumonitis including gender, age (≤67 vs. >67 years, median 67 years), Karnofsky performance score (KPS <90 vs. ≥90), T-category (T1–2 vs. T 3–4), N-category (N0–1 vs. N2–3), M-category (M0 vs. M1), histology (NSCLC vs. SCLC), upfront resection (no vs. yes), upfront systemic treatment (no vs. yes), systemic treatment during or following radiotherapy (no vs. yes), mean radiation dose to ipsilateral lung >20 Gy (no vs. yes), significant cardiovascular disease (no vs. yes), ≥40 pack years (no vs. yes), and history of autoimmune disease (no vs. yes) (Table 2).

### Statistical Considerations

The discriminative power of the symptom-based scoring system was assessed by calculating the AUC, assuming a two-sided significance level of 5%, an AUC of 0.7 and 0.9 under the null and alternative hypothesis (excellent diagnostic accuracy of the scoring system supporting its future use), the statistical power of 90%, and pneumonitis rate of 21.6% at the end of the study (24 weeks after radiotherapy) [7]. Based on these assumptions, 93 patients (20 with pneumonitis) were required within the Full Analysis Set using a two-sided asymptotic test. The Full Analysis Set included all patients who started radiotherapy. An evaluation for the primary endpoint (presence or absence of radiation pneumonitis) was performed in patients available for an assessment who completed at least 75% of questionnaires (paper version of the symptom-based scoring system). Assuming that 5% of patients would not qualify for the Full Analysis Set, 98 patients should be recruited. Calculations were performed with MedCalc software Version 19.1.5 (MedCalc software bv, Ostend, Belgium).

To allow for patient-based analyses, the multiple score values documented for each patient over time were reduced to one clinically relevant, patient-specific value. For patients with pneumonitis, the score at the time of its diagnosis was used, and for patients without pneumonitis the maximum score during the study. In patients who were eligible for the primary endpoint (evaluation of presence or absence of radiation pneumonitis) but died without experiencing pneumonitis prior to completion of the post-radiotherapy follow-up of 24 weeks, the maximum score without pneumonitis was considered. These patient-specific scores represented the fundamental units for further statistical analyses. Sensitivity and specificity were estimated for every possible cut-off value. The ROC curve was used to illustrate the relation between sensitivity and specificity and defined as the plot of sensitivity versus 1-specificity (false-positive rate) across different cut-offs. A ROC-curve corresponding to the greater discriminant capacity of the symptom-based scoring system would be located closer to the upper-left-hand corner, whereas a ROC-curve lying on the diagonal line would reflect the performance of a scoring system not superior to “chance”. AUC was applied as an effective and combined measure of the sensitivity and specificity describing the inherent validity of the usefulness of the score in general. A greater AUC represented better usability of the scoring system. If the AUC is 1, the symptom-based scoring system would be perfect to differentiate between patients with and without pneumonitis. AUC 0.5 would mean that the scoring system is not superior to “chance”. A classification to describe the accuracy is the traditional academic point system: AUC 0.5–0.6 = fail; AUC 0.6–0.7 = poor; AUC 0.7–0.8 = fair, AUC 0.8–0.9 = good, and AUC 0.9–1.0 = excellent [14]. A symptom-based scoring system resulting in an AUC ≤ 0.7 was considered useless. Based on this definition, the following hypothesis system was subjected to statistical analysis: H0: AUC = 0.7 vs. H1: AUC ≠ 0.7.

Non-parametric methods for estimating and testing the AUC using the normal approximation of the asymptotic properties of the AUC with standard errors according to the method of DeLong, DeLong, and Clarke-Pearson were applied [15]. The SAS (SAS Institute Inc., Cary, NC, USA) LOGISTIC procedure with the ROCCONTRAST statement was used to estimate the AUC and its 95% confidence limit and provide the corresponding *p*-value. A two-sided significance level of 5% was pre-specified. If the statistical significance of the AUC is reached, the optimal (most informative) scoring point to predict radiation pneumonitis could be established. The Youden-index (sensitivity + specificity − 1) was used to define the optimal cut-off value represented by the highest value of this index [16,17].

## 3. Results

**Figure 1:**
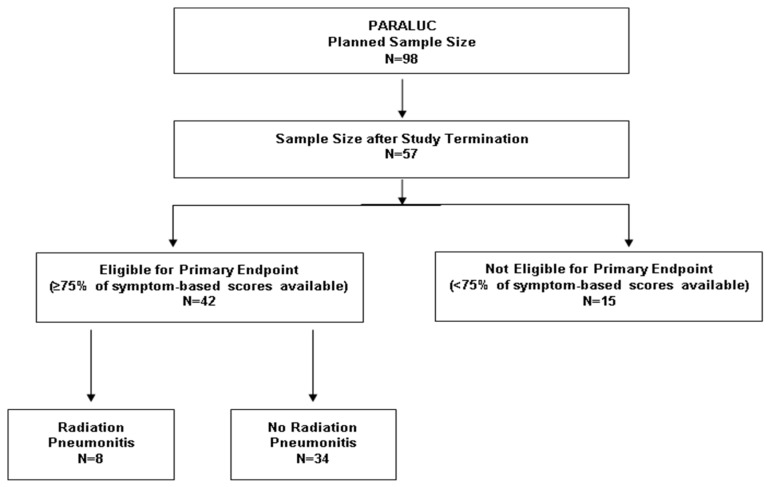
Patient enrollment and eligibility for the primary endpoint (evaluation of the presence or absence of radiation pneumonitis).

The study was closed after 57 of 98 planned patients due to delays caused by the COVID-19 pandemic, longer running time of mandatory pre-studies, and modifications requested by external reviewers (Figure 1). The delay resulted in the expiration of funding. The study was started on 3 November 2020 (first patient in) and closed on 16 June 2022 (last patient out). Of the 57 patients included, 42 patients completed at least 75% of the symptom-based scoring forms and, therefore, were evaluable for the primary endpoint (Figure 1). In these 42 patients, the median total dose was 60 Gy (volumetric modulated arc therapy), and the median dose per fraction was 2.0 Gy. Eleven patients received total doses <60 Gy due to an otherwise unacceptable risk of severe lung toxicity, and two patients neoadjuvant radiotherapy with 50.4 Gy in 28 fractions. Thirty-four patients received concurrent treatment with carboplatin/paclitaxel (*n* = 14), cisplatin/etoposide (*n* = 8), cisplatin/vinorelbine (*n* = 5), cisplatin/pemetrexed (*n* = 3), paclitaxel (*n* = 2), or carboplatin/etoposide (*n* = 2).

Eight of the 42 patients (19%) evaluable for the primary endpoint (presence or absence of radiation pneumonitis) experienced pneumonitis after a median of 6.5 weeks (range: 1–22 weeks) following radiotherapy (Figure 1). The score at the time of pneumonitis was 4 points in one patient, 5 points in six patients, and 6 points in one patient, respectively. Thus, the mean value was 5.00 (±0.50) points. Of the investigated patient and tumor characteristics, significant associations with the development of pneumonitis were found for mean radiation dose to ipsilateral lung >20 Gy (*p* = 0.045) and history of autoimmune disease (*p* = 0.040) (Table 3). In the 34 eligible patients without pneumonitis, maximum scores were 0 points (*n* = 1), 1 point (*n* = 2), 2 points (*n* = 13), 3 points (*n* = 11), and 4 points (*n* = 7), respectively. Thus, the mean value was 2.62 (±0.97) points. In the eight patients who developed pneumonitis, the median increase from baseline score was 3 points (range 2–5 points); only one patient had an increase of <3 points. In the 34 patients without pneumonitis, the median increase was 1 point (0- 4 points); only 2 patients had an increase of ≥3 points. Thus, 7 of 9 patients (78%) with an increase of ≥3 points, and 1 of 33 patients (3%) with an increase of <3 points, respectively, developed pneumonitis (*p* < 0.0001, Fisher’s exact test).

**Figure 2:**
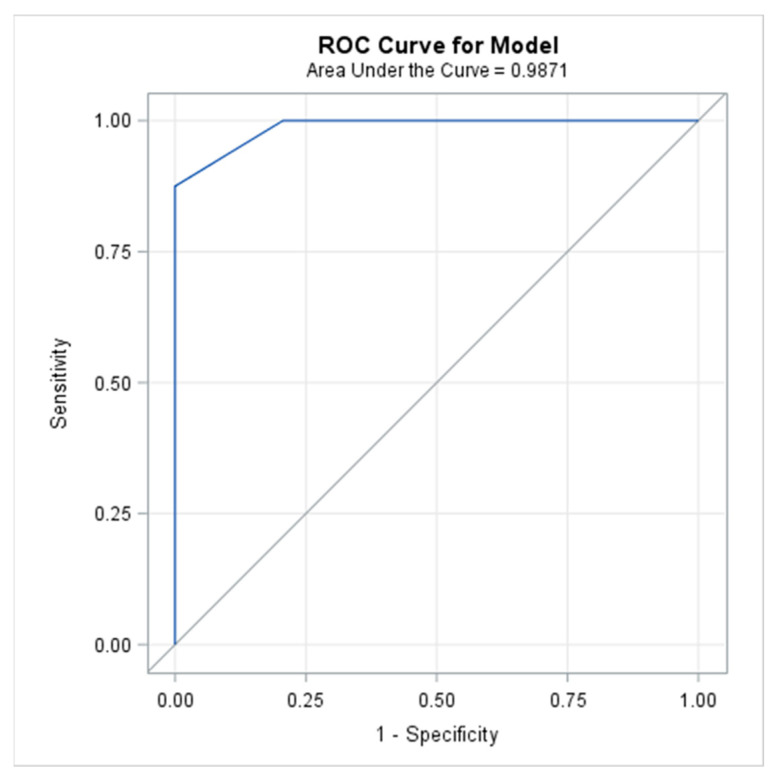
The Receiver Operating Characteristic (ROC) curve used to illustrate the relation between sensitivity and specificity, defined as the plot of sensitivity versus 1-specificity (false-positive rate) across different cut-off values.

The AUC, described with the ROC-curve provides the validity of the usefulness of the symptom-based scoring system; AUC was 0.987 with a 95% Wald Confidence Interval of 0.961–1.000 (Figure 2). Since a greater AUC (maximum possible value = 1.000) represented a higher degree of usefulness of the scoring system to the differentiation between patients with and without pneumonitis, the scoring system provided excellent classifying accuracy. Moreover, the test of whether the AUC was greater than 0.7, yielded high statistical significance with a *p*-value of <0.0001.

This ROC curve indicated a high discriminant capacity of the symptom-based scoring system since it was located close to the upper-left-hand corner. A ROC curve located on the diagonal line would have indicated a poor performance of the scoring system.

Maximum scoring points during the radiotherapy course and the follow-up period ranged between 4 and 6 points (median 5 points) in the 8 patients with pneumonitis and between 0 and 4 points (median 3 points) in the 34 patients without pneumonitis, respectively. Differences between maximum scores and scores at baseline ranged between 2 and 5 points (median 3.5 points) in patients with pneumonitis and between 0 and 4 points (median 1 point) in patients without pneumonitis, respectively. Scores at the time of pneumonitis were 4 points in one patient, 5 points in six patients, and 6 points in one patient, respectively. The highest sensitivity was achieved with scores of 0 to 4 points (100% each), followed by 5 points (87.5%), and the highest specificity with scores of 5 and 6 points (100% each), followed by 4 points (79.4%) (Table 4). The highest PPVs to correctly predict pneumonitis were found for 5 and 6 points (100% each). The NPVs to correctly predict no pneumonitis were high for all points, i.e., 100% for 0–4 points, 97.1% for 5 points, and 82.9% for 6 points, respectively (Table 4). The highest Youden-index was found for 5 points (87.5%), which was considered the optimal cut-off value.

In addition, the 53 patients who completed planned radiotherapy including those receiving planned reduced doses were asked to rate their satisfaction with the symptom-based scoring system. Forty-six patients (87%) completed the questionnaire, and seven patients (13%) refused to complete it without indicating a specific reason. Mean satisfaction scores of individual patients were <4 in 3 patients (6.5%), 4.0–4.9 in 7 patients (15.2%), 5.0–5.9 in 5 patients (10.9%), 6.0–6.9 in 10 patients (21.7%) and 7.0 in 21 patients (45.7%), respectively. Since 43 patients had a mean score of ≥4.0 points, the rate of satisfaction was 93.5%. The mean scores and standard deviations of the three items of the questionnaire are summarized in Table 5.

## 4. Discussion

Pneumonitis is a serious complication after radiotherapy of lung cancer, which can be fatal [2]. Pneumonitis can be delayed and occur only up to five months after completion of the radiotherapy course [3]. After such a long time, symptoms may not be attributed to the previous radiotherapy. As a consequence, radiation pneumonitis will be missed, and patients will not receive appropriate treatment early [4]. This may lead to aggravation of the pneumonitis decreasing the patient’s prognosis [18]. Therefore, it is very important to detect pneumonitis early. An easy-to-use symptom-based scoring system that is able to identify radiation pneumonitis and discriminate this complication from other lung diseases would be helpful. Ideally, this scoring system would be included in an app that can be installed on a patient’s smartphone and used by the patient at home. However, before such an app can be developed, several conditions must be met, including the definition of the scoring point representing the optimal cut-off and patient satisfaction regarding the usability and practicability of the symptom-based scoring system.

The PARALUC trial addressed both aspects. Its primary goal was the identification of the optimal cut-off scoring point. Scoring points observed in this trial during the radiotherapy course and the period of follow-up ranged between 0 and 6. A score of 5 points turned out to be the optimal score to discriminate between patients with and those without pneumonitis. Sensitivity, specificity, and Youden-index were high, namely 87.5%, 100%, and 87.5%, respectively. In addition, the PPV and the NPV were very high. Moreover, the AUC was 0.987, which was close to the maximum possible AUC of 1.000, representing perfect accuracy. When applying the traditional academic point system, the classifying accuracy of our scoring system was “excellent”, i.e., within the highest level of accuracy [14]. In addition, the development of pneumonitis was significantly associated with an increase of ≥3 scoring points compared to the baseline score. Moreover, the patient satisfaction rate regarding the symptom-based scoring system was very high. Thus, the conditions addressed in the PARALUC trial were fulfilled. Since this trial is the first study of its kind, these results cannot be reasonably compared to previous studies.

In addition to the optimal cut-off value and patient satisfaction, 14 factors were evaluated for potential associations with the development of pneumonitis. Of these factors, the mean radiation dose to ipsilateral lung >20 Gy and history of autoimmune disease were significant. Both characteristics were previously reported as risk factors for radiation pneumonitis [5,9,19,20]. Particularly, dose-volume parameters regarding the radiation dose to the lung such as the mean lung dose and the lung volume receiving ≥20 Gy were identified to be associated with pneumonitis [5,9,19,20]. Moreover, in a retrospective study of 169 patients irradiated for lung cancer, a history of chronic inflammatory disease (bronchial asthma, neurodermatitis, rheumatoid arthritis, or psoriasis arthritis) was significantly associated with a greater risk of radiation pneumonitis [20]. The fact that our findings agree with the results of previous studies demonstrates the consistency of our data. However, the limitations of the current trial need to be considered when interpreting its results. Unfortunately, the study was prematurely discontinued without reaching the initially estimated sample size calculated to ensure statistical significance with a probability of 90% (statistical power) when the AUC under the statistical alternative hypothesis is 0.9. In general, power calculations are relevant at the planning stage of a trial and at the analysis stage in case of lack of statistical significance to assess the probability of not having reached statistical significance when the null hypothesis is false. The latter was not applicable in our study since the ROC analysis revealed the statistical significance of the AUC based on the prespecified hypothesis system (*p* < 0.0001) despite the smaller sample size and the fact that only eight patients developed radiation pneumonitis. This was due to an even higher observed AUC (0.987) than initially anticipated. Thus, the study objective was achieved. However, due to the limited sample, it was not possible to validate the optimal cut-off value identified in this trial. Therefore, validation should be performed in a subsequent study. Moreover, one should be aware that there is no specific grading system for radiation-induced pneumonitis, the CTCAE v5.0 system only grades pneumonitis in general [6]. Another factor (procalcitonin) not considered in the PARALUC trial was reported to be helpful in differentiating between acute radiation pneumonitis and bacterial pneumonia [21]. However, procalcitonin is not reasonable for an app used by patients at home.

## 5. Conclusions

In summary, despite the premature termination of this trial, a cut-off value of 5 points was identified as optimal to differentiate between radiation pneumonitis and no pneumonitis with high sensitivity, specificity, and Youden-index. The new symptom-based scoring system provided excellent diagnostic accuracy, and patient satisfaction with the scoring system was high. Thus, important foundations for the development of an app that can be installed on the patient’s smartphone and used by patients at home were laid. However, the optimal cut-off value identified in this trial should be validated in a subsequent study. Moreover, the increased score from the baseline score should be considered in the final version of the app.

## Figures and Tables

**Table 1:** Severity of symptoms and scoring points used for the symptom-based score [7].

Symptom	Severity of Symptoms (Stated by Patients)	Scoring Points
Cough	No	0
	Yes, a little/sometimes	1
	Yes, moderate/regularly	2
	Yes, severe/permanently	3
Shortness of breath	No	0
	Yes, during intense exertion (e.g., climbing stairs)	1
	Yes, during mild exertion (e.g., walking on flat ground)	2
	Yes, at rest	3
Temperature (fever)	No	0
	Yes, between 37.6 and 38.0 °C	1
	Yes, between 38.1 and 39.0 °C	2
	Yes, higher than 39.0 °C	3

**Table 2:** Characteristics of the 42 patients eligible for the primary endpoint (evaluation of presence or absence of radiation pneumonitis).

	Number of Patients (%)
Gender	
Female	14 (33)
Male	28 (67)
Age	
≤67 years	22 (52)
>67 years	20 (48)
Karnofsky performance score	
<90	20 (48)
≥90	22 (52)
T-category	
T 1–2	9 (21)
T 3–4	31 (74)
Not specified	2 (5)
N-category	
N 0–1	14 (33)
N 2–3	27 (64)
Not specified	1 (2)
M-category	
M 0	31 (74)
M 1	11 (26)
Histology	
NSCLC	31 (74)
SCLC	11 (26)
Upfront resection	
No	39 (93)
Yes	3 (7)
Upfront systemic treatment	
No	19 (45)
Yes	23 (55)
Systemic treatment during or after RT	
No	7 (17)
Yes	35 (83)
Mean dose to ipsilateral lung >20 Gy	
No	21 (50)
Yes	21 (50)
Significant cardiovascular disease	
No	24 (57)
Yes	18 (43)
≥40 pack years	
No	23 (55)
Yes	19 (45)
Autoimmune disease	
No	37 (88)
Yes	5 (12)

NSCLC: non-small cell lung cancer; SCLC: small-cell lung cancer; RT: radiotherapy.

**Table 3:** Characteristics of the 42 patients eligible for the primary endpoint, evaluation of presence or absence of radiation pneumonitis, and corresponding pneumonitis rates. *p*-values were calculated with Fisher’s exact test.

	Pneumonitis, *n* (%)	*p*-Value
Gender		0.70
Female	2 (14)	
Male	6 (21)	
Age		0.24
≤67 years	6 (27)	
>67 years	2 (10)	
Karnofsky performance score		1.00
<90	4 (20)	
≥90	4 (18)	
T-category		0.082
T 1–2	4 (44)	
T 3–4	4 (15)	
Not specified	0 (0)	
N-category		0.52
N 0–1	4 (29)	
N 2–3	4 (15)	
Not specified	0 (0)	
M-category		0.66
M 0	7 (23)	
M 1	1 (9)	
Histology		1.00
NSCLC	6 (19)	
SCLC	2 (18)	
Upfront resection		1.00
No	8 (21)	
Yes	0 (0)	
Upfront systemic treatment		1.00
No	4 (21)	
Yes	4 (17)	
Systemic treatment during or after RT		1.00
No	1 (14)	
Yes	7 (20)	
Mean dose to ipsilateral lung >20 Gy		**0.045**
No	1 (5)	
Yes	7 (33)	
Significant cardiovascular disease		0.26
No	3 (13)	
Yes	5 (28)	
≥40 pack years		0.26
No	6 (26)	
Yes	2 (11)	
Autoimmune disease		**0.040**
No	5 (14)	
Yes	3 (60)	

NSCLC: non-small cell lung cancer; SCLC: small-cell lung cancer; RT: radiotherapy; significant *p*-values are given in bold.

**Table 4:** Sensitivities, specificities, Youden-indices, positive predictive values (pneumonitis), and negative predictive values (no pneumonitis) of the different scores ranging between 0 and 6 points.

Cut-Off ≥ Maximum Score	Sensitivity	Specificity	Youden-Index *	Positive Predictive Value ^#^	Negative Predictive Value ^#^
6 points	0.125	1.000	0.125	1.000	0.829
5 points	0.875	1.000	0.875	1.000	0.971
4 points	1.000	0.794	0.794	0.533	1.000
3 points	1.000	0.471	0.471	0.308	1.000
2 points	1.000	0.088	0.088	0.205	1.000
1 point	1.000	0.030	0.030	0.195	1.000
0 points	1.000	0.000	0.000	0.191	1.000

* Youden-index = Sensitivity + Specificity − 1 (to define the optimal cut-off value); ^#^ These values apply only for the prevalence of radiation pneumonitis in this specific data set.

**Table 5:** Patient satisfaction with the symptom-based scoring system. Forty-six patients rated on scales (1–7 points), whether they found the score comprehensible, supportive, and safe. Higher scores represent higher degrees of satisfaction.

Symptom-Based Score Was Considered	Mean Score	Standard Variation
Comprehensible	6.39	1.27
Supportive	5.83	1.74
Safe	5.72	1.82

## Data Availability

Further information regarding this trial is available at clinicaltrials.gov (identifier: NCT04335409).
